# AKT activation by N-cadherin regulates beta-catenin signaling and neuronal differentiation during cortical development

**DOI:** 10.1186/1749-8104-8-7

**Published:** 2013-04-25

**Authors:** Jianing Zhang, Julie R Shemezis, Erin R McQuinn, Jing Wang, Maria Sverdlov, Anjen Chenn

**Affiliations:** 1 Feinberg School of Medicine, Northwestern University, 303 E. Chicago Ave., Chicago, IL, 60611, USA; 2 Department of Pathology, University of Illinois, 909 S. Wolcott Ave. COMRB 6091, Chicago, IL, 60612, USA; 3 Present address: College of Veterinary Medicine and Biomedical Sciences, 1601 Campus Delivery, Colorado State University, Fort Collins, 80523, USA; 4 Present address: Center for Rare and Neglected Diseases, Galvin Life Science Building, University of Notre Dame, Notre Dame, 46556, USA

**Keywords:** Adherens junctions, AKT, β-catenin, ventricular zone, Radial glia, Neuronal differentiation

## Abstract

**Background:**

During cerebral cortical development, neural precursor-precursor interactions in the ventricular zone neurogenic niche coordinate signaling pathways that regulate proliferation and differentiation. Previous studies with shRNA knockdown approaches indicated that N-cadherin adhesion between cortical precursors regulates β-catenin signaling, but the underlying mechanisms remained poorly understood.

**Results:**

Here, with conditional knockout approaches, we find further supporting evidence that N-cadherin maintains β-catenin signaling during cortical development. Using shRNA to N-cadherin and dominant negative N-cadherin overexpression in cell culture, we find that N-cadherin regulates Wnt-stimulated β-catenin signaling in a cell-autonomous fashion. Knockdown or inhibition of N-cadherin with function-blocking antibodies leads to reduced activation of the Wnt co-receptor LRP6. We also find that N-cadherin regulates β-catenin via AKT, as reduction of N-cadherin causes decreased AKT activation and reduced phosphorylation of AKT targets GSK3β and β-catenin. Inhibition of AKT signaling in neural precursors *in vivo* leads to reduced β-catenin-dependent transcriptional activation, increased migration from the ventricular zone, premature neuronal differentiation, and increased apoptotic cell death.

**Conclusions:**

These results show that N-cadherin regulates β-catenin signaling through both Wnt and AKT, and suggest a previously unrecognized role for AKT in neuronal differentiation and cell survival during cortical development.

## Background

In the developing cerebral cortex, neural precursors reside in the ventricular zone (VZ), a neurogenic zone of neuroepithelial cells that lines the lateral ventricles. The specialized microenvironment or niche within the VZ coordinates signaling pathways that regulate the self-renewal and differentiation of neural precursors. Recent studies suggest that the adherens junctions and apical domains of neural precursors contain signaling molecules that regulate interactions between neural precursors critical in maintaining precursor identity [1,2]. Knockdown of the adherens junction proteins N-cadherin [3] or αE-catenin [4] in cortical VZ cells caused reduced β-catenin signaling, increased exit from the cell cycle, premature neuronal differentiation, and increased migration out of the VZ. Although these findings suggest a novel role for N-cadherin-containing junctions in the positive regulation of β-catenin signaling during cortical development, little is known about the molecular mechanisms by which N-cadherin maintains β-catenin signaling.

Recent studies suggest that one way N-cadherin adhesion can influence β-catenin signaling is through regulating the AKT signaling pathway. AKT signaling impacts many fundamental processes regulating growth and metabolism; in organ development, AKT functions in the regulation of normal organ size by mediating signaling downstream of insulin and insulin-like growth factor I [5]. In mammals, the AKT family is comprised of three highly conserved members, AKT1, AKT2, and AKT3. While AKT1^−/−^ mice display a general reduction in sizes of all organs [6], AKT3^−/−^ mice have a selective decrease in brain size [7]. Unlike AKT2 mutant mice, neither AKT1 nor AKT3 mutant mice showed abnormalities in insulin signaling [7], suggesting that brain size regulation by AKT was mediated through alternative non-insulin signaling mechanisms. Recently, findings of active mutations of AKT3 in patients with hemimegalencephaly (HMG), an overgrowth of one hemisphere during brain development, provided further support for the role of AKT signaling in human brain size regulation [8-10].

N-cadherin adhesion can lead to phosphatidylinositol 3-kinase (PI3K)-mediated activation of AKT [11], and activated AKT signaling can stimulate β-catenin signaling. In intestinal stem cells (ISCs), loss of PTEN (phosphatase and tensin homolog), a negative regulator of PI3K/AKT, leads to β-catenin stabilization and upregulated β-catenin signaling [12]. In ISCs the mechanism by which AKT activates β-catenin signaling is via direct phosphorylation of β-catenin at residue Serine 552, which primes β-catenin for 14-3-3ζ binding and stabilization [13]. Moreover, AKT also phosphorylates and inactivates glycogen synthase kinase (GSK)3β at Serine 9; as GSK3β functions as a negative regulator of β-catenin stabilization, AKT phosphorylation of GSK3β can lead to β-catenin stabilization and nuclear β-catenin accumulation [14]. Our previous work suggested that N-cadherin engagement also activates β-catenin signaling through AKT in cortical precursors [3]. While AKT activity was recently described in dividing mouse developing neural precursors [8], how AKT signaling is regulated during neural development, and the underlying mechanisms through which AKT mediates brain growth, remains poorly understood.

The current study seeks to provide greater understanding of the mechanistic relationships between N-cadherin and β-catenin signaling in cortical development, using a combination of *in vivo* and cell culture approaches. Here we find further supporting *in-vivo* data that N-cadherin functions in the cortical VZ to maintain β-catenin signaling. We also find evidence using *in vivo* electroporation approaches and cell co-culture experiments for a cell-autonomous N-cadherin role in receiving Wnt signaling. In addition to its role in transducing Wnt signals through the Wnt co-receptor LRP6, we find that N-cadherin also regulates AKT phosphorylation and activation. Knockdown of N-cadherin leads to reduction of AKT phosphorylation as well as a reduction of Serine 552 phosphorylated β-catenin and Serine 9 phosphorylated GSK3β, both direct targets of active AKT. We show that both β-catenin Ser-552-P and GSK3β Ser-9-P are expressed in mitotic radial glial progenitor cells in the developing cortex, suggestive of activation of AKT signaling in these cells. Using *in utero* electroporation, we show that inhibition of AKT signaling using a dominant negative AKT (DN-AKT) leads to premature exit from the VZ, increased neuronal differentiation, and increased apoptotic cell death. Together, these studies suggest a pathway linking N-cadherin cell adhesion to the regulation of cell survival and differentiation via AKT activation.

## Results

### N-cadherin maintains β-catenin signaling in cortical precursors *in vivo*

During cortical development, β-catenin signaling is active in neural precursors within the VZ [15] and regulates neural precursor proliferation [15,16] and differentiation [17]. Our previous studies suggested that N-cadherin, the primary cadherin of adherens junctions in cortical precursors [18], maintains β-catenin signaling and prevents neural precursors from premature differentiation [3]. Electroporation of N-cadherin-shRNA (Ncad-shRNA) in VZ precursors *in vivo* reduced the expression of an optimized β-catenin signaling reporter, TOPdGFP [3].

**Figure 1:**
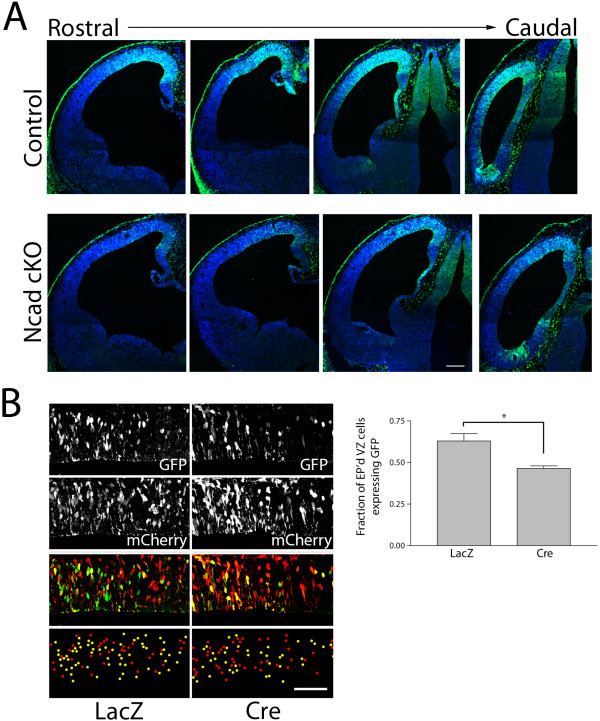
**Conditional knockout of N-cadherin reduces β-catenin signaling in developing cortical precursors.** (**A**) Immunostaining for d2EGFP (green) in E12.0 littermate control (Axin2-d2EGFP; Nes11Cre; Ncad^Flox/+^) and Ncad cKO brain (Axin2-d2EGFP; Nes11Cre; Ncad^Flox/Flox^) embryonic cortex reveals that conditional tissue-wide knockout of N-cadherin leads to reduced EGFP expression in the developing ventricular zone (VZ) (DNA stained with DAPI, pseudocolored blue; bar = 100 µm). (**B**) Focal elimination of N-cadherin reduces β-catenin transcriptional activity. β-catenin mediated transcriptional activation was examined through expression of destabilized GFP controlled by the TOP promoter. E13.5 embryos were electroporated with pTOP-dGFP, pCAG-mCherry, and pCAGLacZ in the control treatment, and with pTOP-dGFP, pCAG-mCherry, and pCAG-Cre in the experimental condition, and analyzed at E14.5. The dot image below shows the positions of the individual electroporated cells (yellow dots represent double-labeled mCherry/dGFP + cells and red dots represent dGFP- cells (expressing mCherry only)). Only electroporated cells in the VZ were included in the analysis, and the proportion of cells expressing dGFP was compared to the total number of electroporated cells in the VZ. *P = 0.0242 by unpaired Student’s *t*-test, n = 3 brains for each. Error bars represent 1 SEM. Bar = 50 µm. EP’d, electroporated.

To confirm the role of N-cadherin in β-catenin signaling in embryonic brains using a genetic conditional knockout approach, we crossed (1) Axin2-d2EGFP mice, which reports endogenous β-catenin signaling by a destabilized EGFP under the control of the endogenous Axin2 promoter/enhancer regions [19,20], with (2) Ncad^Flox/Flox^ mice, in which the first exon of the N-cadherin gene containing the translational start site and upstream transcriptional regulatory sequences are flanked by loxP sequences [21], and (3) Nes11Cre mice, which exhibit widespread Cre recombinase expression in neural progenitor cells by E11 [22]. Staining for d2EGFP in E12.0 Ncad cKO brain (Axin2-d2EGFP; Nes11Cre; Ncad^Flox/Flox^) embryonic cortex and littermate control (Axin2-d2EGFP; Nes11Cre; Ncad^Flox/+^) revealed that conditional tissue-wide knockout of N-cadherin reduced EGFP expression in the developing VZ (Figure 1A).

To examine the cell-autonomous role of N-cadherin in β-catenin signaling in VZ precursors, we co-electroporated expression plasmids for Cre recombinase and TOPdGFP [23] into the VZ of E13.5 Ncad^Flox/Flox^ embryos. This approach enables conditional deletion of genes from cells receiving the Cre plasmid [4,15,24] and, as the reporter GFP is produced by the simultaneously introduced plasmids, the signaling readout is not affected by historical activation of the signaling pathway. Staining for GFP 24 hours after electroporation showed that, compared to cells electroporated with the pcDNA-lacZ control, Cre electroporation reduced β-catenin signaling (Figure 1B). Together, these results support our previous finding that cell-autonomous N-cadherin is required for maintenance of β-catenin signaling in cortical neural progenitor cells.

### N-cadherin functions in Wnt-induced β-catenin signaling in 293 T cells in a cell-autonomous manner

We observed previously that N-cadherin adhesion regulated endogenous β-catenin signaling activity in neural precursors [3]. The most well-characterized and common activator of β-catenin signaling is the family of secreted Wnt proteins. Wnt signaling results in inactivation of a phosphodestruction complex that normally serves to target free cytosolic β-catenin for ubiquitin-mediated degradation [25]. Our previous observations that increasing neural precursor cell density could increase β-catenin transcriptional activation [3] activity suggested that cell proximity/density could regulate Wnt/β-catenin signaling.

**Figure 2:**
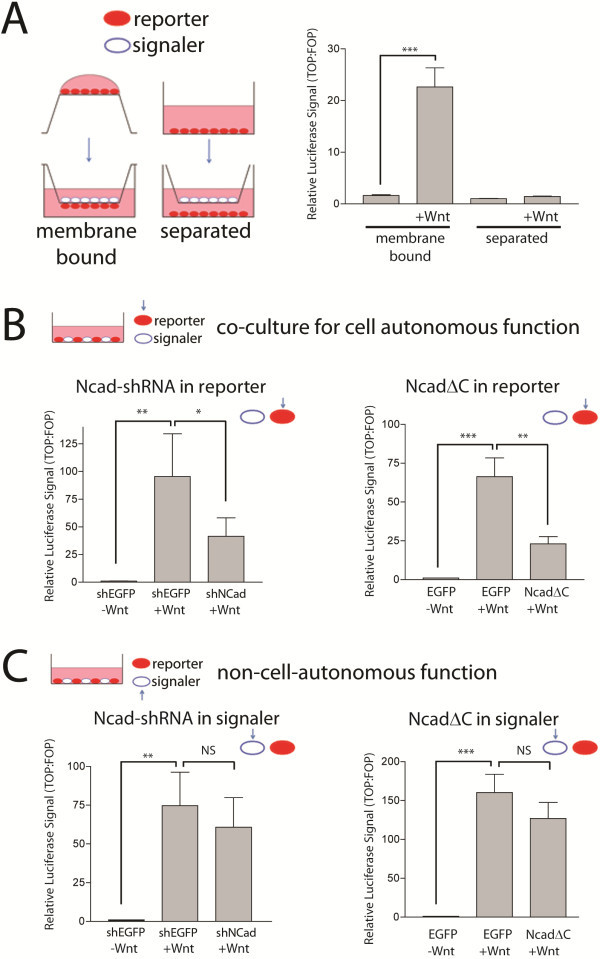
**N-cadherin functions in Wnt-induced β-catenin signaling in a cell-autonomous manner.** (**A**) Separate populations of 293 T cells were transfected with Wnt3a expression and pSuper8TOPFlash reporter constructs and plated either on the opposite of a polycarbonate transwell filter (“membrane bound”) or on the bottom of the transwell assay (“separated”). In a TOPFlash reporter assay, Wnt3a-expressing signaler cells induced β-catenin signaling in reporter cells adherent on the opposite face of the transwell membrane, but not in reporter cells plated separately at the bottom of the chamber. *P* = 0.004 by repeated measures analysis of variance (ANOVA), ***P < 0.001, Neuman Keuls *post-hoc* test, n = 3. (**B**) Reduction of N-cadherin by shRNA (Ncad-shRNA) or by overexpression of C-terminal truncated N-cadherin (NcadΔC) in Wnt-responsive cell results in reduced Wnt-activated β-catenin transcriptional activation. Wnt3a transfected signaler cells were co-cultured with pTOPflash-transfected reporter cells co-transfected with either shRNA to N-cadherin (*P* = 0.0012 by repeated measures ANOVA, **P < 0.01, *P < 0.05 by Neuman Keuls *post-hoc* test; n = 4) or NcadΔC (*P* = 0.0006 by repeated measures ANOVA, ***P < 0.001, **P < 0.01 by Neuman Keuls *post-hoc* test; n = 3), and luciferase activity was measured 24 hours after co-culture. (**C**) Inhibition of N-cadherin in the Wnt-producing signaling cell does not affect Wnt-mediated β-catenin signaling. Wnt3a transfected signaler cells were co-transfected with either Ncad-shRNA (*P* = 0.0024 by repeated measures ANOVA, **P < 0.01 by Neuman Keuls *post hoc* test; n = 4) or NcadΔC (*P* = 0.0002 by repeated measures ANOVA, ***P < 0.001 by Neuman Keuls *post-hoc* test; n = 3), co-cultured with pTOPflash-transfected reporter cells, and luciferase activity was measured 24 hours after co-culture.

To examine further the relationship of cell proximity and Wnt/β-catenin signaling, we created signaler (Wnt “signaler”) and reporter (Wnt “reporter”) cells (that report activation of β-catenin-mediated transcription) by transfecting separate populations of 293 T cells with Wnt3a expression and pSuper8xTOPFlash reporter constructs and examined signaling in cells plated either on the opposite of a polycarbonate transwell filter (“short distance”) or on the bottom of the transwell chamber (“long-distance”) (Figure 2A). When plated on opposite sides of the filter, cells can make physical contacts with the cells on the opposite side through the 0.4 µm pores [26,27]. We found that Wnt3a-expressing signaler cells induced β-catenin signaling in reporter cells adherent on the opposite face of the transwell membrane, but not in reporter cells plated separately at the bottom of the chamber (Figure 2A). Together with our previous findings that N-cadherin mediates density-dependent β-catenin signaling, this observation that Wnt induction of β-catenin signaling requires close cell-cell apposition suggested a role for N-cadherin cell adhesion in mediating Wnt signaling.

To examine specifically the role of N-cadherin in Wnt signaling, we conducted loss-of-function studies of N-cadherin in either signaler or reporter cells. Transfection of Ncad-shRNA or dominant negative N-cadherin with a C-terminal β-catenin binding domain truncation (N-cadΔC) in reporter cells reduced Wnt-induced β-catenin signaling in a cell-autonomous fashion (Figure 2B). In contrast, N-cadherin knockdown or expression of N-cadΔC in signaler cells resulted in a trend towards reduced Wnt-induced β-catenin signaling (Figure 2C), suggesting only a modest role for non-cell-autonomous function for N-cadherin in Wnt signaling. Together, the *in vivo* electroporation and co-culture findings suggest that N-cadherin maintains Wnt-induced β-catenin signaling through a cell-autonomous mechanism. These co-culture findings also suggest a role for non-autonomous N-cadherin in Wnt signaling, likely mediated in part by the requirement for close cell apposition.

### Reduction of N-cadherin leads to reduction of AKT and LRP6 phosphorylation

Recent studies have suggested that cadherin adhesion can both positively [28] and negatively [25] regulate β-catenin signaling in a Wnt-independent manner. To explore further the downstream pathways linking cell-autonomous N-cadherin function to β-catenin signaling, we examined targets of Wnt-independent and Wnt-stimulated activation of β-catenin following knockdown or blockade of N-cadherin in cultured cells.

**Figure 3:**
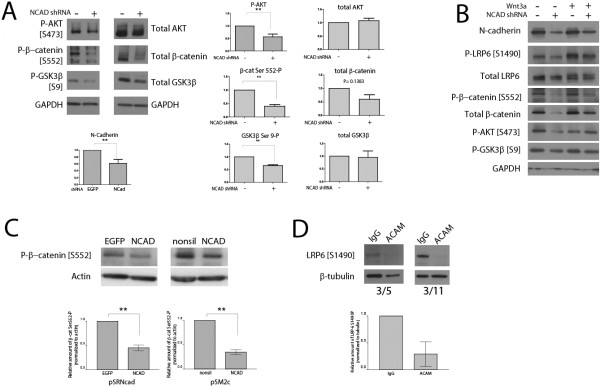
**Reduction of N-cadherin leads to reduction of AKT and LRP6 phosphorylation.** (**A**) Transfection of N-cadherin-shRNA (Ncad-shRNA) reduced the amount of AKT phosphorylated at Serine 473 (P-AKT [S473P]) and AKT targets β-catenin phospho-Serine 552, β-catenin [S552-P]) and phosphorylated GSK3β (P-GSK3β [Ser 9]). N-cadherin, *P* = 0.0151 by paired *t*-test, n = 7; P-AKT [S473], *P* = 0.0182 by paired *t*-test, n = 6; P-β-catenin [S552], *P* = 0.0083 by paired *t*-test, n = 3; P-GSK3β [Ser 9], *P* = 0.0085 by paired *t*-test, n = 3. Consistent with the stabilizing role of phosphorylated Ser 552, there was a trend for reduction in total β-catenin in N-cad-shRNA transfected cells (*P* = 0.1383 by paired *t*-test, n = 2). (**B**) Cells stably expressing N-cad-shRNA knockdown reveal reduced levels of AKT phosphorylation (P-AKT [S473], phospho-GSK3β (P-GSK3β [S9]), and phospho-β-catenin (P-β-catenin [S552]). N-cadherin knockdown also reduced baseline phosphorylation of LRP-6 (P-LRP6 [S1490]) as well as Wnt3a-induced phosphorylation of LRP6. (**C**) Primary mouse cortical progenitors were nucleofected with two different shRNA to N-cadherin or EGFP and cell extracts were blotted for phospho-β-catenin Serine 552 (P-β-catenin [S552]). In both cases, N-cadherin knockdown resulted in reduced phosphorylation of β-catenin at Ser 552. shRNA1: *P* = 0.0119 by paired *t*-test (n = 3); shRNA2: *P* = 0.0055 by paired *t*-test (n = 3). (**D**) Primary mouse neural stem cells derived from E14.5 mouse cortices were treated for 24 hours with either control IgG or N-cadherin function blocking antibody 20 µg/ml, and cell extracts were blotted for phospho-LRP6 [S1490]. Both biological replicates are shown.

To examine the role of N-cadherin in regulation of β-catenin in the absence of Wnt stimulation, we transfected 293 T cells with N-cadherin-shRNA or EGFP-shRNA control. Western blot analysis after 24 hours incubation showed that, in the absence of exogenous Wnt, Ncad-shRNA reduced active AKT (phospho-AKT Ser 473). We also found reductions in two direct downstream targets of active AKT - GSK3β phosphorylated at Serine 9, and β-catenin phosphorylated at Serine 552 - following N-cadherin-shRNA transfection (Figure 3A). Phosphorylation of β-catenin at Ser 552 increases its transactivation by increasing its stability [13], while phosphorylation of GSK3β leads to inactivation of the GSK3β and can result in accumulation of β-catenin [14]. We also observed a trend where Ncad-shRNA reduced total β-catenin levels, consistent with prior observations that phosphorylation at Ser 552 increasing β-catenin stability [13]. 

In 293T cell lines stably expressing Ncad-shRNA, we observed similar findings: reduction of N-cadherin expression led to reduced levels of active AKT (phosphor-Ser 473), GSK3β phospho-Ser 9, and β-catenin phosph-Ser 552 in the absence of Wnt (Figure 3B). Together, these findings suggest that, in the absence of Wnt, N-cadherin can regulate AKT activity and β-catenin levels via phosphorylation of β-catenin at Ser 552.

In the absence of exogenous Wnt, we also observed in the stable N-cadherin knockdown lines that levels of phosphorylated LRP6, the Wnt co-receptor, were also reduced (Figure 3B). As phosphorylation of LRP6 results from activation by Wnt [29], together with the observations that Wnt stimulation of signaling required N-cadherin (Figure 2), this finding suggested that Wnt-stimulated β-catenin signaling via LRP6 is impacted by N-cadherin levels. When the stable N-cadherin knockdown cell lines were transfected with Wnt3a to activate the Wnt signaling pathway, we observed increased phosphorylation of LRP6, as expected. We then found that, in N-cadherin knockdown cells, Wnt3a-induced phosphorylation of LRP6 was also reduced. Together, these findings suggest that N-cadherin levels also regulate Wnt-induced phosphorylation of its co-receptor LRP6.

To examine the role of N-cadherin in Ser 552 phosphorylation of β-catenin in cortical neural stem cells (NSC), we utilized mouse cortical NSC derived from E14.5 mouse cortices [30,31]. Using two different shRNA constructs against N-cadherin (pSRNcad (“shRNA1”) and pSM2cNcad (“shRNA2”)), we found that reduction in N-cadherin caused a reduction in the amount of β-catenin phosphorylated at Ser 552 (Figure 3C). To examine the role of N-cadherin in the phosphorylation and activation of the Wnt co-receptor LRP6 in NSC, we treated the NSC with either IgG control antibody or N-cadherin function-blocking antibodies (GC-4, Sigma; St. Louis, USA) and examined levels of phosphorylated LRP6 (Figure 3D). We found that inhibition of N-cadherin dramatically reduced the levels of phospho-LRP6 in primary cortical NSC, providing further support for the notion that N-cadherin also functions in β-catenin signaling in neural stem/progenitors by mediating both Ser 552 phosphorylation of β-catenin and LRP6-mediated Wnt signaling.

### Targets of AKT activity are expressed in dividing radial glia during cortical development

**Figure 4:**
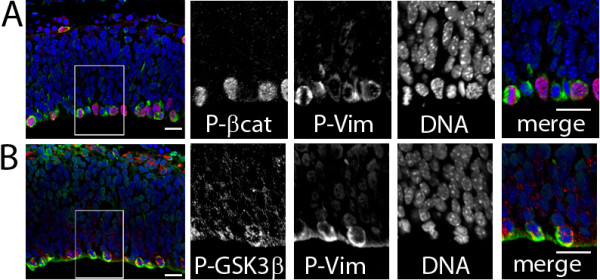
**Targets of AKT activity are expressed in dividing radial glia during cortical development.** Immunohistofluorescence staining of cortical sections at embryonic day 12.5 reveals AKT activity as assessed by targets of AKT phospho-β-catenin [S552] and phosphor-GSK3β [S9] in the ventricular zone (VZ). (**A**) Phospho-beta-catenin-Ser 552 (pseudocolored red) is expressed in dividing radial glial cortical precursors identified by the expression of phosphorylated vimentin 4A4 (P-vim, pseudocolored green) located at the apical (lumenal) surface of the VZ. (**B**) Phospho-GSK3beta-Ser 9 (pseudocolored red) is expressed in dividing radial glial cortical precursors expressing phosphorylated vimentin 4A4 (P-vim, pseudocolored green) at the apical ventricular surface. Scale bar = 20 µm. 74.3±5.9% (1 SEM) of P-Vim expressing cells were also expressing P-β-catenin (n = 3 brains), and 82.5±3.6% (1 SEM) of P-Vim-expressing cells were also P-GSK3β positive (n = 3 brains).

These findings that N-cadherin functions in Ser 55 phosphorylation of β-catenin in cortical NSC suggest that AKT signaling may play an important role in cerebral cortical development. A recent study pointed to somatic activating mutations in AKT3 that caused HMG and highlighted the presence of phosphorylated AKT (P-Ser-473) in dividing cortical neural precursors suggestive of active AKT signaling [8]. To examine downstream targets of AKT signaling in the developing cortex, we examined the expression of GSK3β-Ser 9-P and β-catenin Ser 552-P in VZ precursors. We observed widespread localization of GSK3β Ser 9P and β-catenin Ser 552P in dividing apical neural progenitors in the VZ. P-GSK3β and P-β-catenin co-localized with the radial glial marker phospho-Vimentin 4A4 [8,32] (Figure 4). 74.3±5.9% (1 SEM) of P-Vim expressing cells were also expressing P-β-catenin, and 82.5±3.6% (1 SEM) of P-Vim-expressing cells were also P-GSK3β positive. While GSK3β Ser 9 and β-catenin Ser 552 have been shown to be direct targets of AKT signaling [12], our data do not rule out other kinases that might phosphorylate these residues (e.g. protein kinase C (PKC) and GSK3β Ser 9 [33], PKA and β-catenin Ser-552 [34]). However, together with the observations of active AKT in these same cells, our findings provide further support for active AKT signaling in radial glial precursors in the developing cortex.

### AKT maintains β-catenin signaling and neural differentiation *in vivo*

**Figure 5:**
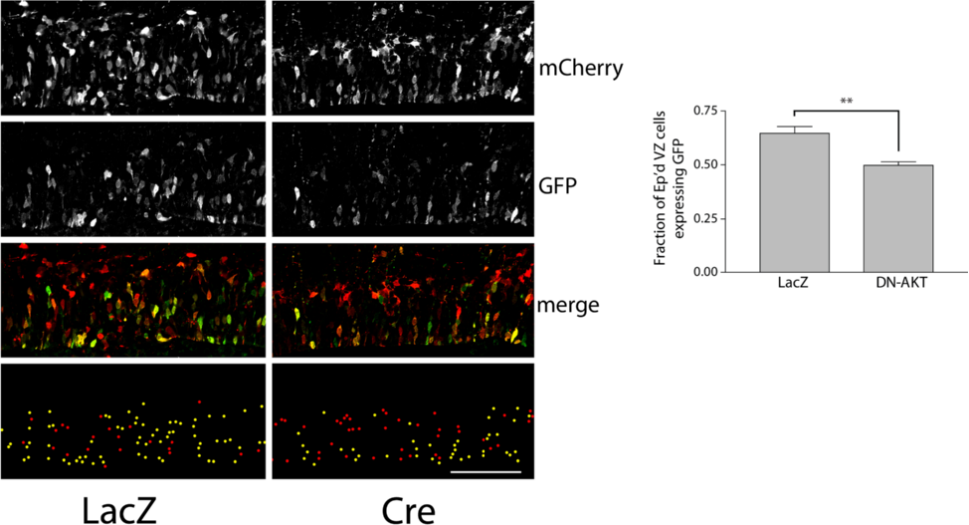
**Inhibition of AKT signaling reduces β-catenin transcriptional activity*in vivo*.** β-catenin mediated transcriptional activation was examined through expression of destabilized GFP controlled by the TOP promoter. E13.5 embryos were electroporated with pTOP-dGFP, pCAG-mCherry, and pcDNA in the control treatment, and with pTOP-dGFP, pCAG-mCherry, and dominant negative AKT (DN-AKT) in the experimental condition and analyzed 30 hours after electroporation. The dot image below shows the positions of the electroporated cells (green dots represent electroporated cells co-expressing dGFP and mCherry, and red dots represent dGFP- cells (expressing mCherry only)). Only electroporated cells in the ventricular zone (VZ) were included in the analysis, and the proportion of cells expressing dGFP was compared to the total number of electroporated cells (red, labeled by mCherry) in the VZ. Compared to pcDNA control, DN-AKT expression reduced the fraction of TOPdGFP-expressing cells from 73 to 53% (n = 3 brains each), **P = 0.0043 by unpaired *t*-test. Error bars represent 1 SEM. Scale bar = 100 µm. EP’d, electroporated.

To examine directly whether AKT regulates β-catenin signaling in cortical progenitors *in vivo*, we inhibited AKT signaling by *in utero* electroporation of a kinase-dead dominant negative AKT (DN-AKT) and examined β-catenin signaling using a co-electroporated β-catenin signaling reporter, TOPdGFP. We found that DN-AKT reduced β-catenin signaling in cortical VZ cells, suggesting that AKT is required for maintaining β-catenin signaling during cortical development (Figure 5). As β-catenin signaling is both necessary and sufficient to drive cortical precursor self-renewal, we next sought to examine whether modulating AKT signaling influenced precursor identity.

**Figure 6:**
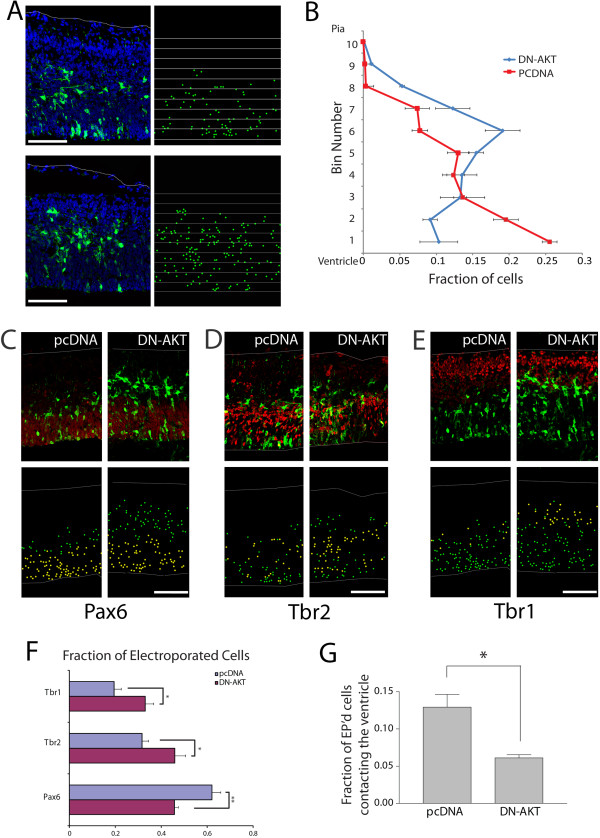
**AKT prevents premature differentiation of neural progenitors*in vivo*.** (**A**) E13.5 forebrains were electroporated *in utero* with pCAG-GFP and 4-fold (by mass) excess of pcDNA control or K197M dominant negative AKT (DN-AKT) and analyzed at E14.5 (n = 4 brains each). Electroporated cells were identified using antibody staining against GFP and sections counterstained with DAPI. The pial surface is indicated by the white line. Scale bar = 100 µm. (**B**) To quantify changes in cortical positioning of electroporated cells, ten equal sized bins were drawn over each image covering the cortical plate. Each dot corresponds with the soma of an electroporated cell. The fraction of the total GFP + cells was then graphed. The *x*-axis denotes the fraction of the total number of electroporated cells in each bin. Brackets indicate 1 SEM. (**C**-**F**) Sections of electroporated brains were stained for Pax6 (**C**), Tbr2 (**D**), and Tbr1 (**E**). Electroporated cells are green, and the respective antigens are red. Bar = 100 µm. The dot plots show the electroporated cells co-expressing the marker as yellow and electroporated cells not expressing the marker as green. (**F**) Cell histograms show the fraction of electroporated cells that express each marker after electroporation (yellow/yellow + green dots). DN-AKT causes premature neuronal differentiation as defined by the alterations in Tbr1, Tbr2, and Pax6 expression. For Pax 6, DN-AKT versus control (n = 4 brains for each, **P = 0.0071), Tbr2 (n = 2 for DN-AKT, n = 4 for pcDNA control, *P = 0.0406), and Tbr1 (n = 4 for each, *P = 0.0277), unpaired *t*-test. Error bars represents SEM. Scale bar = 100 µm. (**G**) DN-AKT increases the fraction of cells that lose contact with ventricular surface. n = 4 brains, *P = 0.0368, paired *t*-test. Error bar represents SEM. EP’d, electroporated.

AKT signaling regulates normal organ size during development: AKT1^−/−^ mice display a general reduction in sizes of all organs [6]; AKT3^−/−^ mice present a selective decrease in brain size [7]; and three recent studies suggest that mutations and overactivation of AKT3 can cause enlargement of the brain in cases of human HMG [8-10]. Consistent with the idea that AKT activity regulates neural precursor proliferation, retroviral overexpression of AKT1 promoted retention of cortical progenitors in the VZ and subventricular zone and increased NSC self-renewal [35]. After *in utero* electroporation of DN-AKT, we observed increased cell exit from the VZ towards the pial surface and premature neuronal differentiation (Figure 6). AKT signaling may also function in maintaining cell adhesion, as inhibition by DN-AKT decreases the fraction of cells in contact with the ventricle (Figure 6G).

**Figure 7:**
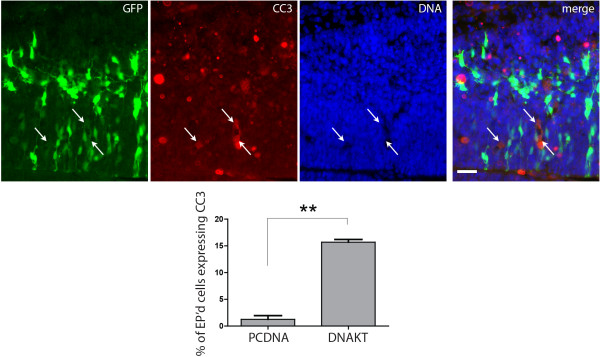
**Dominant negative AKT increases apoptotic cell death*in vivo.*** E13.5 forebrains were electroporated *in utero* with pCAG-GFP and 4-fold (by mass) excess of pcDNA control or K197M dominant negative AKT (DN-AKT) and analyzed at E14.5. Sections of electroporated brains were stained for GFP, cleaved caspase 3, and DNA stained with DAPI. Arrows point to electroporated cells that are expressing cleaved caspase 3. Bright red round spots are non-specific staining not associated with cells. Bar = 50 µm. Compared with control, electroporation of DN-AKT causes increased cell death, *P* = 0.0067 by paired students *t*-test. n = 3 brains for each condition. EP’d, electroporated.

AKT signaling normally impacts a broad range of cellular processes including apoptotic cell death. Many members of the apoptotic signaling cascade are targets of AKT-mediated phosphorylation and inactivation [36], and GSK3β inhibition by AKT has been shown to protect both neurons [37] and neural precursor cells from apoptosis [38]. To examine whether inhibition of AKT signaling in the developing cortex alters cell apoptosis, we co-stained *in utero* electroporated cortices for the apoptotic marker cleaved caspase 3. We found that compared with control GFP-electroporated cells, where only ~1% of electroporated cells were apoptotic, a much larger fraction of cells that received DN-AKT (~15%) exhibited signs of apoptosis (cleaved caspase 3 expression, condensed nucleus by DAPI) (Figure 7). These findings suggest that a critical role of AKT signaling in cortical development is in maintenance of cell survival during the neurogenic period. Further study is required to determine whether different threshold levels of AKT signaling may regulate the decisions to differentiate versus undergo programmed cell death in cortical development.

## Discussion

**Figure 8:**
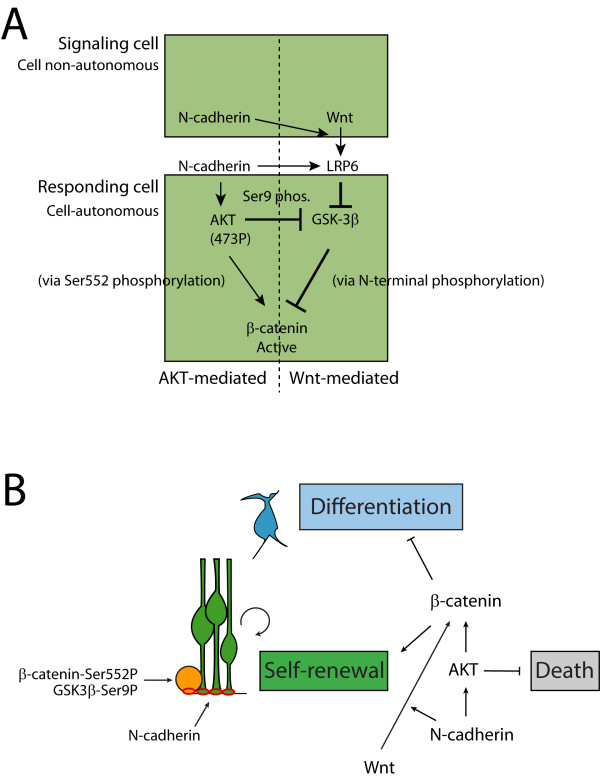
**Model for the function of N-cadherin in β-catenin activation and cortical development.** (**A**) N-cadherin functions in the activation of β-catenin signaling via AKT-mediated phosphorylation of β-catenin and Wnt-mediated activation of the Wnt co-receptor, LRP6. In Wnt-mediated signaling, N-cadherin is required in a cell-autonomous manner in the responding cell and is involved in signaling via phosphorylation of the Wnt co-receptor LRP6. N-cadherin also functions to stimulate β-catenin signaling via AKT activation and subsequent phosphorylation of β-catenin at Ser 552. (**B**) In cortical radial glial neural progenitors, N-cadherin regulates epithelial integrity, and enhances Wnt-mediated and AKT-mediated β-catenin activation. Downstream of N-cadherin, AKT signaling functions to promote self-renewal via activation of β-catenin signaling as well as inhibiting apoptosis.

A growing body of work points to a critical role for adherens junctions in maintaining neural progenitor identity [39], and a recent study provided evidence that regulation of N-cadherin expression by Foxp2 and Foxp4 initiates neuronal delamination from the VZ and differentiation [40]. Here we expand our understanding of the molecular mechanisms through which N-cadherin regulates the neural precursor niche in cerebral cortical development. Our observations suggest that cell-autonomous N-cadherin functions in both Wnt-mediated and AKT-mediated β-catenin transcriptional activation. Furthermore, our findings suggest that N-cadherin mediated activation of AKT signaling functions in cortical precursors *in vivo* to promote β-catenin signaling, and inhibition of AKT leads to reduced β-catenin signaling and premature exit from the VZ, neuronal differentiation, and increased apoptosis (Figure 8).

The Cre-mediated N-cadherin knockout in developing cortical precursors and the co-culture experiments presented provide two complementary lines of evidence suggesting that reduction or interference with N-cadherin reduces β-catenin signaling activity in a cell-autonomous manner. Our findings with co-cultured signaling/responder cells also suggest - but do not provide definitive evidence for - a role for N-cadherin-dependent cell contact in Wnt-signaling. Our observations of a small trend of reduced Wnt-signaling when N-cadherin is reduced in Wnt-producing cells are consistent with mechanisms where cell-cell adhesion enhances Wnt signaling, potentially by optimizing the physical interactions and/or proximity of ligand and receptor. *In vivo*, our studies are also consistent with a function for N-cadherin in physical retention of neural precursors in the VZ. However, our findings that cells still located in the VZ also show reduced β-catenin signaling (FiguresÂ1B and Figure 7[4]) suggest that the model that N-cadherin maintenance of β-catenin occurs solely via physical retention of cells in the VZ is likely overly simplistic. We find that when N-cadherin or αE-catenin (another critical component of the VZ adherens junction) [4] are reduced, we observe reductions in β-catenin signaling that likely precede exit from the VZ, as electroporated cells that have not yet left the VZ still exhibit reduced signaling.

Distinguishing between a purely adhesive versus signaling role of N-cadherin is challenging; loss of N-cadherin will cause breakdown of cell contacts and disruptions of neuroepithelial adhesion [41,42], and the possibility remains that adhesion and signaling function of N-cadherin cannot be separated. It is well known that regulators of cell polarity (such as scribble [43,44] and aPKC [45,46]) play a critical role in inhibiting neoplastic growth, and we believe that N-cadherin maintenance of cell adhesion in neural precursors may similarly link growth and polarity, but via control of β-catenin signaling and adhesion in a normal developmental context.

How cadherin adhesion can both negatively and positively regulate β-catenin signaling activity remains poorly understood. Observations that the forced overexpression of cadherins antagonizes β-catenin transcriptional activation suggested the simple model that cadherins set a threshold for β-catenin signaling. However, our findings suggest that N-cadherin functions both in Wnt-mediated activation of β-catenin via phosphorylation of the Wnt co-receptor LRP6 and in activation of β-catenin via AKT-mediated phosphorylation of Ser 552 of β-catenin. In contrast to the widely held viewpoint that cadherins are considered negative regulators of β-catenin signaling through sequestration [47], our findings lend additional support to a growing body of studies suggesting a positive role for a classical cadherin in β-catenin signaling [28,48-51].

Our findings that N-cadherin functions to positively regulate Wnt-mediated β-catenin signaling through the Wnt co-receptor LRP6 support a recent study showing that Casein kinase 1 (CK1) association with LRP5/6 requires cadherin [51]. p120 catenin, via interactions with cadherin and CK1, appears to control assembly of the Wnt signaling complex [50]. As phosphorylation of LRP6 by CK1 isoforms plays an essential function in Wnt receptor complex activation [52], these findings together support a positive role for cadherin in Wnt/β-catenin signaling.

While Wnt-mediated β-catenin signaling in cortical development has been suggested by LRP6 mutant mice [53] and Wnt overexpression studies [54], the role of alternative regulation of β-catenin signaling in cortical development is not well investigated. Here, our evidence suggests that AKT regulates β-catenin signaling in cortical precursors *in vivo*, and inhibition of AKT activity leads to premature neuronal differentiation and exit from the VZ. Consistent with previously reported functions of AKT signaling in cell survival, we also observed that inhibition of AKT *in vivo* by electroporation leads to increased apoptotic cell death.

The alterations we observe in the proportions of cells that are progenitors or differentiated neurons after AKT inhibition may be a consequence of increased cell death in progenitor populations. However, it is difficult to extrapolate the effects of increased progenitor cell death on cells that differentiate from the progenitors, as their number would also be reduced as a consequence of progenitor death. Our findings of a relative increase in Tbr1 and Tbr2-expressing cells as well as increased migratory distance following DN-AKT supports a role in differentiation versus an exclusive role in cell survival.

Our results support recent findings that AKT and PI3K signaling functions in cortical growth, with gain-of-function mutations implicated in human HMG [8-10], and suggest that abnormal activation of β-catenin signaling may underlie the molecular mechanisms driving cortical overgrowth in HMG.

## Conclusions

Here, we provide evidence that cell-autonomous N-cadherin co-ordinates both Wnt-dependent and AKT-mediated β-catenin activation. In the presence of Wnt, N-cadherin regulates LRP6 phosphorylation, while when Wnt is absent, N-cadherin also regulates β-catenin transcriptional activation via AKT-mediated phosphorylation of β-catenin Ser 552. Using *in utero* electroporation approaches, we found evidence that AKT regulates β-catenin signaling levels in VZ cortical precursors, and loss of function experiments showed that inhibition of AKT signaling causes increased apoptosis, premature neuronal differentiation, and exit from the VZ. Our findings that AKT functions in cortical precursors support the observations of recent studies suggesting a role for AKT in human megalenecephaly.

## Methods

### Animals

All mice in this study were treated according to protocols reviewed and approved by the institutional animal care and use committees of Northwestern University, Animal Study Protocol # 2010–1863, approved by the Northwestern University Office for the Protection of Research Subjects Institutional Animal Care and Use Committee. Timed-pregnant C57BL/6 mice were ordered from Charles River Laboratories (Wilmington, MA, USA). Ncad^Flox/Flox^ mice (B6.129S6(SJL)-*Cdh2*^*tm1Glr*^/J) and Nes-Cre (B6.Cg-Tg(Nes-cre)1Kln/J) mice were obtained from The Jackson Laboratory (Bar Harbor, ME, USA). Ncad^Flox/Flox^ mice were genotyped using primers 5′-TGCTGGTAGCATTCCTATGG-3′ and 5′-TACAAGTTTGGGTGACAAGC-3′ as previously described [21]. Nes-Cre mice were genotyped according to The Jackson Laboratory protocols available at http://jaxmice.jax.org/protocolsdb/f?p=116:2:903366808665603::NO:2:P2_MASTER_PROTOCOL_ID,P2_JRS_CODE:288,003771 (Cre). Axin2-d2EGFP reporter mice [19] (a gift from F Costantini, Columbia University Medical Center, New York, NY, USA) have been used to report β-catenin signaling *in vivo*[20]. Axin2-d2EGFP reporter mice were genotyped as described [19].

Axin2-d2EGFP and Nes-Cre mice were mated to Ncad^Flox/Flox^ mice to generate (Axin2-d2EGFP; Ncad^Flox/+^) and (NesCre; Ncad^Flox/+^) F1 progenies, respectively. (Axin2-d2EGFP; Ncad^Flox/+^) and (NesCre; Ncad^Flox/+^) F1 progenies were mated to generate E12.0 embryos of (Axin2-d2EGFP; NesCre; Ncad^Flox/Flox^) genotype. Littermate embryos of (Axin2-d2EGFP; NesCre; Ncad^Flox/+^) and (Axin2-d2EGFP; Ncad^Flox/Flox^) genotypes were used as controls without N-cadherin conditional knockout. Embryos were harvested at E12.0.

### Plasmids

pSUPER.retro.puro driving shRNAs against N-cadherin (GACTGGATTTCCTGAAGAT; nucleotides 431–449 of mouse N-cadherin [GenBank:AB008811], which is identical to nucleotides 215–233 of human N-cadherin [GenBank:BC036470]) and control EGFP (CGATGCCACCTACGGCAAG; nucleotides 786–804 of GFP [GenBank:U55762]) were generously provided by M Wheelock (University of Nebraska, Lincoln, NE, USA). The shRNA to EGFP was used as the control for the Ncad-shRNA construct. pcDNA-Myr-AKT and DN-AKT (K197M) were kindly provided by Anna Kenney (Memorial Sloan Kettering cancer center 1275 York Ave., New York City, NY USA). The shRNA to eGFP was used as the control for the pSUPER Ncad-shRNA construct. Similar phenotypes were obtained with a second shRNA construct targeting mouse N-cadherin; mouse shRNAmir to N-cadherin in pSHAG-MAGIC 2c (pSM2c) retroviral vector was obtained from Open Biosystems (Thermo Scientific; 81 Wyman St. Waltham, MA 02454), accession number NM_007664, with oligonucleotide ID: V2MM_13176, target sequence: GCAGGCAAAGTTCCTGATATA. Control non-silencing shRNA (cat. RHS1703) is a negative control that expresses sequence TCTCGCTTGGGCGAGAGTAAG which has no homology to known mammalian genes. This non-silencing construct was used as a control to the two shRNAmir constructs.

### *In utero* electroporation

For *in utero* injection, timed-pregnant mice at E13.5 were anesthetized using inhalation of isoflurane mixed in a constant ratio with oxygen, after which abdominal fur was removed, and the uterine horns were exposed through a midline laparotomy incision. DNA solution (2.5 µL) in H_2_O containing 0.0125% fast green was injected through the uterine wall into the lateral ventricle of the embryos using a glass micropipette made from a microcapillary tube. After injection, Tweezer-trodes (BTX, 84 October Hill Rd. Holliston, MA 01746–1388) were applied across the outside of the uterus, oriented to flank the embryonic brain, and five 50 ms square pulses of 39 V with 950 ms intervals were delivered by an electroporator (BTX 830). Following injection and electroporation, the uterus is returned inside the abdomen and the abdominal muscle wall and skin sealed with sutures.

To study the effect of N-cadherin knockdown on TOP-dGFP signaling *in vivo*, 0.8 µg/µL of DNA was used for pcDNA-Cre, pcDNA-lacZ, pCAG-mCherry and TOP-dGFP. To study the effect of DN-AKT on TOP-dGFP signaling *in vivo*, 0.25 µg/µl of pCAG-mCherry, 0.5 µg/µl of TOP-dGFP, and 1.0 µg/µl of DN-AKT or pcDNA3.0 were used. For migration and differentiation studies, 0.25 µg/µl of DNA for pCAG-GFP and 0.75 µg/µl of DN-AKT or pcDNA3.0 were used.

For the TOP-dGFP signaling studies, embryos were sacrificed 30 hours after electroporation. For the distribution and differentiation studies, embryos were sacrificed 24 hours after electroporation. Brains were fixed for 8 to 16 hours in 4% paraformaldehyde, cryoprotected in 30% sucrose dissolved in PBS overnight, and embedded in OCT (Optimal Cutting Temperature compound). A cryostat was used to make 12 µm coronal sections.

For studies of TOPdGFP expression in Ncad^Flox/Flox^ mice, histograms in Figure 1B were derived from three brains, 312 cells (mCherry, pcDNA-lacZ control), and three brains, 261 cells (mCherry, pcDNA-Cre). To determine the effect of DN-AKT on TOPdGFP in C57/B6 mice, three brains, 522 cells (mCherry, DN-AKT), and three brains, 645 cells (mCherry, pcDNA3.0 control), were analyzed. Cell distribution histograms were derived from four brains (GFP, DN-AKT), 706 cells, and four control brains (GFP, pcDNA3.0), 540 cells.

For studies of cell identity, histograms of Pax6, Tbr2 and Tbr1 expressing cells were derived from four brains, 690 GFP + cells (Pax6, DN-AKT), four brains, 1099 GFP + cells (Pax6, pcDNA3.0); two brains, 227 GFP + cells (Tbr2, DN-AKT), four brains, 489 GFP + cells (Tbr2, pcDNA3.0); four brains, 652 GFP + cells (Tbr1, DN-AKT), and four brains, 1092 GFP + cells (Tbr1, pcDNA3.0).

### Immunohistochemistry and tissue analysis

Brain sections were incubated with blocking solution (5% goat serum and 0.3% Triton X-100 in PBS) for 1 hour at room temperature, and then incubated with primary antibodies diluted in blocking solution for 2 hours at room temperature (for Pax6, Tbr2, Tbr1, mCherry and TOPdGFP) or overnight at 4°C (for Axin2-d2EGFP, phospho-AKT, phospho-GSK3β, phospho-β-catenin, phospho-Vimentin 4A4, and GFP in distribution assay). Primary antibodies used were anti-GFP chicken polyclonal antibody (1:1000, Abcam, 1 Kendall Sq, Cambridge, MA 02139), anti-N-cadherin mouse monoclonal antibody (1:1000, BD Transduction LaboratorieBD Biosciences 2350 Qume Drive San Jose, CA 95131), anti-DsRed (mCherry) rabbit polyclonal antibody (1:1000, Clontech 1290 Terra Bella Ave. Mountain View, CA 94043 USA), anti-Pax6 mouse monoclonal antibody (1:200, Developmental Studies Hybridoma University of Iowa, Department of Biology 028 Biology Building East Iowa City, Iowa 52242–1324), anti-Tbr2 rabbit polyclonal antibody (1:250, Abcam), anti-Tbr1 rabbit polyclonal antibody (1:200, Chemicon EMD Millipore Headquarters 290 Concord Road Billerica, MA 01821), anti-phospho-AKT Ser 473 D9E rabbit monoclonal antibody (1:50, Cell Signaling 3 Trask Lane Danvers, MA 01923), anti-phospho-GSK3β Ser 9 rabbit polycolonal antibody (1:100, Cell Signaling), and anti-phospho-β-catenin Ser 552 rabbit antibody (1:250, kindly provided by Mark Hembree and Linheng Li, Stowers Institute 1000 E 50th St, Kansas City, MO 64110 **U**). After washing in PBS, the sections were then incubated with secondary antibodies and DAPI diluted in PBS for 1 hour at room temperature. Secondary antibodies used were Alexa488-conjugated goat anti-chicken IgG antibody (1:1000, Molecular Probes Life Technologies 3175 Staley RoadGrand Island, NY 14072 USA), Alexa555-conjugated goat anti-mouse IgG antibody (1:1000; Molecular Probes), Alexa555-conjugated goat anti-rabbit IgG antibody (1:1000; Molecular Probes), Alexa647-conjugated goat anti-mouse IgG antibody (1:1000; Molecular Probes), Alexa647-conjugated goat anti-rabbit IgG antibody (1:1000; Molecular Probes), and Alexa488-conjugated goat anti-mouse IgG antibody (1:1000; Molecular Probes). Finally, immunofluorescent images of the (Ax2GFP; NesCre; Ncad^Flox/Flox^) brains and controls were captured using a Nikon ECLIPSE TE2000-U fluorescent microscope (Nikon Inc. 1300 Walt Whitman Road Melville, NY 11747–3064, U.S.A.). Confocal images were captured using a Zeiss UV LSM510 confocal microscope Carl Zeiss Microscopy, LLC One Zeiss Drive Thornwood, NY 10594 USA). The analysis throughout the paper was carried out in a semi-blinded manner as previously described [3].

### Primary neural stem cell culture, shRNA, and treatment with function-blocking antibodies

For Western blots of N-cadherin, phospho-β-catenin S552, β-catenin, phospho-Akt S473 and Akt, E14.5 mouse primary cortical cultures were generated from E14.5 mouse embryos using minor modifications to the method described in [55]. After dissection, cells were disassociated with 0.25% Trypsin/EDTA (Sigma), washed by DMEM with 10% FBS, and resuspended in clone density media (CDM) containing DMEM (Cellgro Mediatech, Inc. 9345 Discovery Blvd. Manassas, VA 20109), 2 mM L-glutamine (Cellgro), 1 mM N-Acetyl-cysteine, 1 mM sodium pyruvate, and B27 and N2 supplement (final 1×) with 25 ng/mL FGF-2. Five million primary cortical precursors were transfected with 10 µg shRNA constructs against N-cadherin or EGFP control using Nucleofector following the manufacture’s protocol (Lonza Lonza Inc. 90 Boroline Road Allendale, NJ 07401). Transfected cells were plated on Poly-D Lysine treated 6-well tissue culture plates and allowed to recover in the described media (as above) at 37°C for 24 hours before lysing for Western blots. Adherent cortical NSC cultures were generated from E14.5 mouse embryos as described in [30,31]. After dissection, cells were dissociated with Accutase (Millipore EMD Millipore Headquarters 290 Concord Road Billerica, MA 01821), washed three times with NS-A medium (Stem Cell Technologies #5750 STEMCELL Technologies Inc. 570 West Seventh Avenue, Suite 400, Vancouver, BC, V5Z 1B3, Canada), rinsed once more with NSC medium (NS-A supplemented with FGF-2, EGF, N-2A, B27, L-glutamine, Sodium pyruvate, N-acetyl cysteine, and Penicillin-streptomycin as in [31]) and plated on laminin-coated (treated with 2 µg/cm^2^ laminin) dishes. To obtain material for protein analysis, 5 million mouse E13.5 or E14.5 primary cortical precursors was transfected by AMAXA Nucleofection (Amaxa Biosystems, Gaithersburg, MD, USA) following manufacturer protocols; cells were allowed to recover in the described media (as above) at 37°C for 24 hours before lysing for Western blots. Cells were plated at 1.8 × 10^5^/cm^2^ for 24 hours, then treated with either function-blocking N-cadherin antibody (ACAM, GC-4, Sigma) or control IgG1κ isotype control (BD Pharmingen cat. 554721 BD Biosciences 2350 Qume Drive San Jose, CA 95131) (both at 20 µg/ml, final concentration) for 24 hours.

### 293 T co-culture and luciferase assays

293 T cells were maintained in cDMEM media containing DMEM (Cellgro), 10% FBS, 2 mM L-glutamine (Cellgro) and Penicillin:streptomycin (Cellgro). For transwell co-culture assays, signaler and reporter cells were transfected separately on day 1 and plated together after 24 hours. On day 1, signaler cells (1 × 10^6^ 293 T cells) were transfected with 1.0 µg Wnt or pcDNA3.0 control using Lipofectamine 2000 following the manufacturer’s protocol (Life Technologies Life Technologies 3175 Staley RoadGrand Island, NY 14072 USA) and plated in a 24-well plate. Reporter cells (5 × 10^5^ 293 T cells) were transfected with 1.0 µg Super8xTOP FLASH Luciferase and 0.1 µg Super8xFOP Renilla using Lipofectamine 2000, and plated either (1) on the outside of the 6.5 mm transwell membrane inserts with 0.4 µm pore size (Costar Corning Incorporated Life Sciences 836 North Street Building 300 Suite 3401 Tewksbury, MA 01876, USA) inverted in a 6-well plate or (2) on the bottom of a 12-well plate. After 24 hours, the 6.5 mm transwell membrane inserts with reporter cells attached were inverted and placed in a 12-well plate soaked in media. Signaler cells in the 24-well plate were lifted by 0.25% Trypsin/EDTA and one-quarter of the cells were plated on the inside of the transwell membrane inserts (“Membrane-bound” condition). The pore size of these inserts is 0.4 µm, through which cells plated on opposite sides of the insert can establish physical contacts [26,27]. For the “Separated” condition, the signaler cells were plated inside transwell membrane inserts and placed above the 12-well plate with the reporter cells plated on the bottom of the well [26]. After incubated for 24 hours at 37°C, the reporter cells were lysed for Dual luciferase assay (Dual-Luciferase® Reporter Assay System, Promega Promega Corporation 2800 Woods Hollow Road Madison, WI 53711 USA).

For 293 T co-culture with N-cadherin knockdown, the reporter and signaler cells were generated similarly to that described above (1 × 10^6^ cells were transfected by 1.0 µg Super8xTOP FLASH Luciferase and 0.1 µg Super8xFOP Renilla as reporter cells, and a further 1 × 10^6^ cells were transfected by 1.0 µg Wnt3a or pcDNA3.0 control as signaler cells), with the difference that 0.25 µg N-cadherin/EGFP-shRNA (or NCAD-ΔC/pIRES-GFP control) were transfected into either the reporter or signaler cells; pcDNA3.0 empty vector was used to bring the total DNA mass to 1.6 µg in all the transfections. After 24 hours, the reporter and signaler cells were lifted by 0.25% Trypsin/EDTA, and one-half of the signaler cells and one-sixth of the reporter cells were mixed and plated on a 24-well plate. The cells were lysed after another 48 hours for Dual luciferase assay.

### 293 T transfection

To collect the protein lysates for N-cadherin knockdown studies, 293 T cells were transiently transfected using Lipofectamine 2000 following the manufacturer’s protocol (Life Technologies). 293 T cells (1 × 10^6^) were plated on a 12-well tissue culture plate and transfected by 1.2 µg Wnt or pcDNA3.0 control together with 2.0 µg N-cadherin or EGFP shRNA construct. Cells were allowed to recover in cDMEM at 37°C for 24 hours before lysing for Western blots. For AKT studies, 1 × 10^6^ 293 T cells were plated on a 24-well tissue culture plate and immediately transfected by 1.0 µg HA-AKT-K179M or pcDNA3.0 control.

### Western blot analysis

To prepare whole cell lysates, 293 T cells or mouse NSC lines were obtained as described above and collected in RIPA lysis buffer (50 mM Tris–HCl, pH 7.4, 150 mM NaCl, 1% TritonX-100, 1% sodium deoxycholate, 0.1% SDS) containing Protease Inhibitor Cocktail Set III (Calbiochem EMD Millipore Headquarters 290 Concord Road Billerica, MA 01821) and Phosphotase Inhibitor Cocktail I (Sigma). Cells were passed through an insulin syringe to shear and centrifuged at 14,000 rpm for 10 minutes at 4°C. Supernatants were collected and combined with 4× SDS sample buffer and boiled for 5 to 10 minutes. The resulting protein samples were applied to 8% or 6% SDS-PAGE gel and transferred to PVDF membranes. The blots were blocked with 5% milk/TBS-T or 5%BSA/TBS-T for 1 hour and probed with the following primary antibodies: rabbit anti-phospho-LRP6 Ser 1490 (1:1000 dilution in 5% BSA; Cell Signaling), rabbit anti-LRP6 (1:1000 dilution in 5% milk; Cell Signaling), mouse anti-N-cadherin (1:1000 dilution in 5% milk; BD Transduction Laboratories), rabbit anti-phospho-β-catenin Ser 552 (1:1000 dilution in 5% BSA; Cell Signaling), rabbit anti-β-catenin (1:1000 dilution in 5% milk; Cell Signaling), rabbit anti-phospho-AKT Ser 473 (1:1000 dilution in 5% BSA; Cell Signaling), rabbit anti-AKT (1:1000 dilution in 5% milk; Cell Signaling), rabbit anti-phospho-GSK3β Ser 9 (1:1000 in 5% BSA; Cell Signaling), rat (rabbit) anti-GSK3β (1:500 dilution in 5% milk; Calbiochem), mouse anti-actin (1:1000; dilution in 5% milk; Chemicon), and rabbit anti-GAPDH (1:1000; dilution in 5% milk; Santa Cruz Biotechnology Santa Cruz Biotechnology, Inc. 10410 Finnell Street Dallas, Texas 75220 U.S.A.). Blots were washed × 3 with TBS-T before incubation with HRP-conjugated goat anti-mouse antibody (1: 3000 dilution in 5% milk, Bio-Rad 2000 Alfred Nobel Drive, Hercules, CA 94547) or HRP-conjugated goat anti-rabbit antibody (1: 5000 dilution in 5% milk, Bio-Rad) for 1 hour at room temperature. The signal was visualized using the ECL Western blotting system (Amersham GE Healthcare Biosciences P.O. Box 643065 Pittsburgh, PA 15264–3065). To ensure equal loading, protein concentrations were determined using the BCA Protein Assay (Pierce Thermo Scientific; 81 Wyman St. Waltham, MA 02454). Densitometry of scanned films was performed with ImageJ.

## Abbreviations

CDM: clone density media; CK1: Casein kinase 1; DMEM: Dulbecco’s modified Eagle’s medium; DN-AKT: dominant negative AKT; EDTA: Ethylenediaminetetraacetic acid; EGFP: enhanced green fluorescent protein; FBS: fetal bovine serum; GFP: green fluorescent protein; GSK: glycogen synthase kinase; HMG: hemimegalencephaly; ISC: intestinal stem cell; N-cadΔC: N-cadherin with a C-terminal β-catenin binding domain truncation; Ncad-shRNA: N-cadherin-shRNA; NSC: neural stem cells; PBS: phosphate-buffered saline; PI3K: phosphatidylinositol 3-kinase; PKC: protein kinase C; shRNA: small hairpin RNA; VZ: ventricular zone.

## Competing interests

The authors declare that they have no competing interests.

## Authors’ contributions

JZ performed the experiments shown in Figures 1, 2, 3, 4, 5 and 6, and contributed to experimental design and manuscript writing. JA and EM designed and generated data in the experiments shown in Figure 2, and JA contributed to manuscript writing and editing. JW generated data in Figures 2B,C. MS performed experimental replicates for western blot protein quantification and cleaved caspase 3 staining in Figure 7. AC provided overall guidance for experimental design and wrote the manuscript. All authors read and approved the final manuscript.
